# Wing Base Structural Data Support the Sister Relationship of Megaloptera and Neuroptera (Insecta: Neuropterida)

**DOI:** 10.1371/journal.pone.0114695

**Published:** 2014-12-11

**Authors:** Chenjing Zhao, Xingyue Liu, Ding Yang

**Affiliations:** Department of Entomology, China Agricultural University, Beijing, China; Australian Museum, Australia

## Abstract

The phylogenetic status and the monophyly of the holometabolous insect order Megaloptera has been an often disputed and long unresolved problem. The present study attempts to infer phylogenetic relationships among three orders, Megaloptera, Neuroptera, and Raphidioptera, within the superorder Neuropterida, based on wing base structure. Cladistic analyses were carried out based on morphological data from both the fore- and hindwing base. A sister relationship between Megaloptera and Neuroptera was recovered, and the monophyly of Megaloptera was corroborated. The division of the order Megaloptera, the traditional higher classification, into Corydalidae (Corydalinae + Chauliodinae) and Sialidae, was also supported by our wing base data analyses.

## Introduction

Megaloptera is a holometabolous insect order belonging to the superorder Neuropterida (lacewings and allies), and generally considered to be among the most archaic holometabolous insects [1]. Currently, there are ca. 380 extant described species of Megaloptera in the world, divided into two families, Corydalidae (dobsonflies and fishflies) and Sialidae (alderflies), both widely distributed in all zoogeographical realms. Although the taxonomy of Megaloptera is well studied [2]–[4], the phylogenetic status of Megaloptera within Endopterygota (Holometabola) is controversial.

Traditionally, Megaloptera and Raphidioptera have been considered sister taxa. This viewpoint was recently advocated by Beutel et al. [5] in the holometabolan context, based on morphological data. This study proposed two synapomorphies for Megaloptera + Raphidioptera, the distinctly flattened larval head, and the prognathous adult head [5]; both of which, however, are considered to be symplesiomorphic by Aspöck and Haring [6]. The sister relationship of Megaloptera + Raphidioptera was also proposed in the context of the evolution of the female postabdomen [7]. However, the ovipositor of Raphidioptera members can be clearly deduced from the primitive archaeognathan pattern, and only the extreme elongation of the ninth gonocoxites and the complete fusion of the eighth gonapophysis are derived conditions [8]. Megaloptera + Raphidioptera was also supported by molecular phylogenetic evidence based on six single-copy nuclear protein-coding genes [9].

In contrast, a sister relationship between Megaloptera and Neuroptera was first proposed by Aspöck et al. [10] in a phylogenetic analysis of Neuropterida based on morphological data, and was later corroborated by Aspöck and Aspöck [8] after homologization of the genitalic sclerites of Neuropterida. Four synapomorphies supporting Megaloptera + Neuroptera are: (1) elongation of the larval stipites, (2) integration of the larval cardines into the head capsule, (3) a complex organization of trichobothria on the ectoproct in form of a rosette, and (4) the ninth male gonocoxites becoming the appendices of tergite 9 [8]. Moreover, the sister relationship of Megaloptera + Neuroptera is also supported by 170 morphological characters in combination with eight nuclear and mitochondrial gene fragments [11]. Furthermore, mitochondrial genomic data strongly support the grouping of Megaloptera + Neuroptera [12], [13].

Despite the undisputed monophyly of Raphidioptera and Neuroptera, the monophyly of Megaloptera also remains to be clarified. Nonetheless, most studies based on both morphological and molecular data recover a monophyletic Megaloptera [8], [13], [14]. The proposed synapomorphies supporting the monophyly of Megaloptera are: (1) the presence of eversible sacs in the fused male gonocoxites 11, (2) the distal fusion of subcosta and radius veins, and (3) the presence of abdominal lateral gills in the first instar larvae [3], [8]. However, in many megalopteran species the male gonocoxites 11 do not have eversible sacs, and the distal fusion of subcosta and radius veins is also present in many neuropteran species. Furthermore, the monophyly of Megaloptera was questioned by Stys and Bilinski [15], based on the similar telotrophic ovaries shared by Sialidae and Raphidioptera. Later studies by Büning [16], [17] disagree, concluding that the ovaries are not suitable synapomorphic traits owing to “several switches between polytrophic and telotrophic ovaries” having occurred during the radiation of ancient insect taxa. A comprehensive Neuropterida phylogenetic analysis using a molecular dataset, as well as a molecular plus morphological dataset, suggests a paraphyletic Megaloptera [18], in which either Corydalidae or Sialidae is assigned as the sister group of all the remaining Neuropterida. In summary, these contradictory results to date do not allow for an unambiguous conclusion regarding the monophyly of Megaloptera.

The insect wing is a complex system composed of membranes, veins, folding and flexion lines, and marginal setae. The combination of all these elegant structures is what provides insects with the capability of flight [19]. Because the acquisition of wings is considered to be a significant morphological innovation, uncovering the origin and evolution of insect wings is important to understand the evolution and diversification of insects [1]. The origin of insect wings and flight has been studied from many different views, such as the morphology of extant [20], [21] and fossil [22], [23] and functional morphology [24], [25]. In addition, the wing base structure also plays an important role in insect flight. This structure is a complicated system composed of the notal margin, axillary sclerites, median plates, and vein base, as well as of articulations and folding lines formed by these structures [26]. The wing base structure transmits flight power from the thorax to the wing veins. Moreover, the rotation of the wing is controlled directly by muscles inserted into the wing base sclerites [19]. The presence of many sclerites and articulations in the wing base area provides robust evidence for topological correspondence [27], allowing the identification of homology in the sclerites. Notably, various reliable landmarks guarantee homologization of the wing base sclerites, even between distantly related orders [28]–[31]. These wing base structural characteristics allow for the selection of a large quantity of qualitative data, and are expected to contain much useful information for uncovering the deep phylogeny of the group. This character system evolves very slowly, probably because of functional restrictions implicit in complex flying and wing folding mechanisms [32]. Therefore, the insect wing base structure has attracted attention as a source of deep phylogenetic information [31].

In this study we examined and described the forewing and hindwing base structure of the three orders of Neuropterida: Megaloptera, Neuroptera, and Raphidioptera. Our comparative morphological study adds to overall understanding of the diversification of the neuropteridan wing base. A phylogenetic analysis among these three orders was performed based on a morphological character dataset including both the fore- and hindwing base structural data. Our results shed new light on the higher phylogeny of Neuropterida, especially the phylogenetic status and monophyly of Megaloptera.

## Materials and Methods

### Ethics statement

No specific permits were required for the insects collected for this study. The specimens were collected by using light trap and sweeping net. The field studies did not involve endangered or protected species. The species herein studied are not included in the “List of Protected Animals in China”.

### Taxa examined

Ingroup taxa included three families/subfamilies of Megaloptera, nine families of Neuroptera, and two families of Raphidioptera (S1 Table). Tenthredinidae of Hymenoptera and Amphipsocidae of Psocoptera were selected as outgroups, because Hymenoptera was recently considered to be the sister of all the remaining holometabolous insects, and Psocoptera is a member of Paraneoptera, which is generally considered to be the sister group of Holometabola [1]. The wing base structure was observed using a ZEISS Stemi 2000-c stereoscope (Carl Zeiss, Jena, Germany). Wing bases are highly three-dimensional structures; therefore, the wing was stretched artificially upwards for observation. In addition, all figures were made in dorsal view of the wing base. All specimens examined were initially preserved in 80% ethanol in the field after collection, and transferred to −20°C for long-term storage upon the arrival at the Entomological Museum of China Agricultural University (CAU), Beijing.

### Terminology

**Figure 1:**
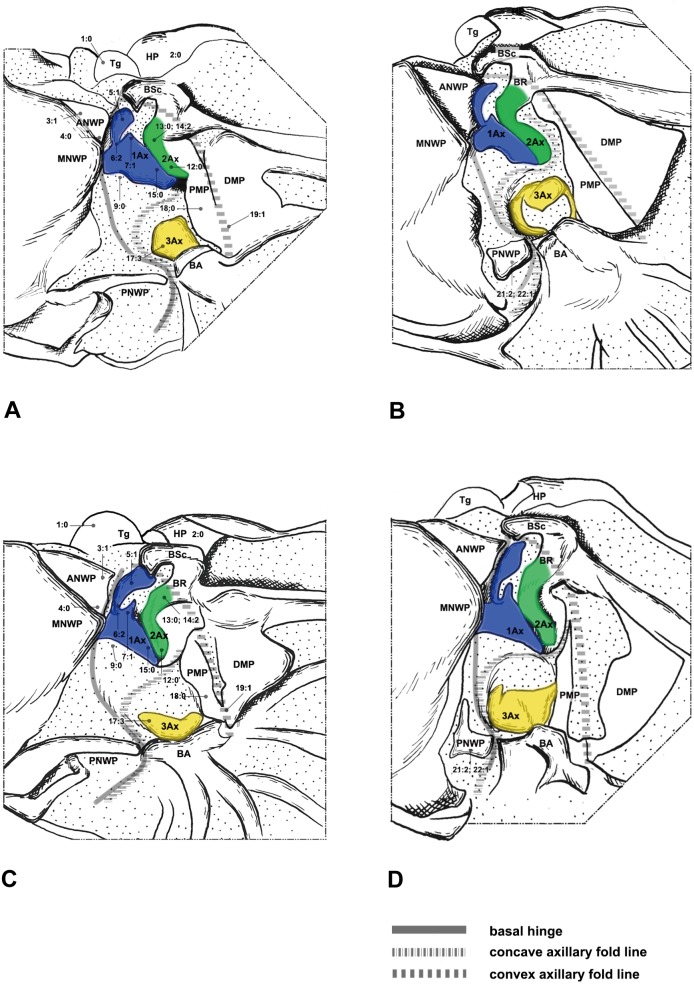
Wing base of Corydalidae (Megaloptera). (A) *Protohermes costalis* Walker (Corydalinae), forewing base; (B) same, hindwing base; (C) *Neochauliodes punctatolosus* Liu & Yang (Chauliodinae), forewing base; (D) same, hindwing base. Number of morphological character: character state for phylogenetic analysis is indicated by straight line for relevant position.

The terminology of the wing base sclerites follows that of Brodsky [19] and Matsuda [33]. The terminology of the folding lines follows that of Wootton [26]. The following abbreviations are used in the text and figures: 1Ax–4Ax = first, second, third, and fourth axillary sclerites; ANWP, MNWP and PNWP = anterior, median and posterior notal wing processes; BA = basanale, BR = basiradiale; BSc = basisubcostale; HP = humeral plate; Tg = tegula; PMP and DMP = proximal and distal median plates.

### Phylogenetic analysis

Twenty-three characters from the fore- and hindwing base structure were coded (see result: Character description of wing base structures used for phylogenetic analysis). Quantitative characters were not coded unless variation could be coded clearly and was not continuous. As mentioned by Yoshizawa and Saigusa [34], Yoshizawa [28], [29] and Ninomiya and Yoshizawa [31], fore- and hindwing base structures usually show analogous modifications when both wings are homogeneous in shape. To prevent any particular character from being double-counted, data should generally be selected from the forewing or hindwing alone. However, in our Neuropterida study, the PNWP of the hindwing base are significantly different from those of the forewing base. Hence, selecting data from both wings is justified. A detailed discussion of the PNWP of the hindwing follows.

First, the 4Ax are connected firmly to the basal part of the PNWP or notum through a less sclerotized region in Neuropterida, rather than the membrane in the hindwing base. Second, whether we used the basal hinge or concave axillary fold line as a landmark, both of the posterior parts of the two lines run between the distal end of 4Ax and the proximal tip of 3Ax. Therefore, we consider that 4Ax belongs to and presents as a distally detached part of the PNWP. Herein, we use the detached part of the PNWP as the term instead of 4Ax. The similar sclerite was observed in Zoraptera and Embioptera, but its homology is arguable because of different landmarks [28]. Also, differentiation of the sclerite, which is homologous with the distal part of the PNWP, was reported in Mecoptera [32]. Because the detached sclerite was not observed in the outgroups, it is the feature most clearly different from the plesiomorphic condition, and can be regarded as an apomorphy of Neuropterida.

Each family was treated as a terminal taxon for ingroups and outgroups in phylogenetic analysis. The dataset (including 19 forewing base characters and four hindwing base characters) was analyzed in NONA ver. 2.0 [35] using heuristic parsimony, with 100 replications [multiple TBR + TBR (mult*max*)]. Bootstrap values were calculated using 10000 replicates and a general heuristic search; branches with bootstrap values of ≤50% were collapsed. Bremer’s decay indices were calculated with TNT ver. 1.1 [36] and WinClada ver. 1.00.08 [37]. Unambiguous characters were mapped using WinClada. The data matrix is shown in S2 Table. The dataset were also analyzed in PAUP*4.0b10 [38] to test and verify and calculate the CI and RI of each character, using heuristic parsimony analysis, with 100 random stepwise additions of taxa (tree-bisection-reconnection (TBR) branch swapping) under ACCTRAN optimization, characters unordered and of equal weight and MulTrees option in effect.

## Comparative Morphology of the Wing Base Structures

### General morphology of wing base structure

#### Folding lines

There are five folding lines in the axilla: basal hinge, convex axillary fold line, concave axillary fold line, convex axillary flexion line, and anterior axillary fold line [26]. These lines have been widely used as principal landmarks for identifying the homology of wing base structures [28], [31].

#### Notum and articulation

The notum has three principal wing processes: ANWP, MNWP, and PNWP. The apex of the ANWP is almost always adjacent to the anteroproximal margin of the head and neck of 1Ax, forming the headmost articulation between the notum and axillary region along the basal hinge. The MNWP articulates with the proximal margin of the body of 1Ax. The PNWP extends from the posterolateral corner of the notum, and is a tapering process in general, articulating with the proximal tip of 3Ax along the basal hinge.

#### Axillary region

The axillary region consists of three single axillary sclerites (1Ax, 2Ax, and 3Ax), two median plates (the PMP and DMP), and some of the basal sclerites of veins (the HP, BSc, BR, and BA predominantly).

Three regions are generally recognized in the 1Ax, the head, neck, and body [32]. The head and neck regions are usually much narrower than the body. The body of 1Ax is generally subtriangular and articulates proximally with the MNWP. 1Ax and the PNWP are generally widely separated. The head of 1Ax cranially articulates with the proximal tip of BSc. 1Ax and BR are usually closely approximated although 2Ax often occurs between them.

The 2Ax is a slender sclerite located just distal to 1Ax, which articulates with 1Ax along the convex axillary flexion line. Anteriorly, 2Ax, which is ridged and broadened, is closely associated with the BR. In addition, the BR is fused to it, and the fused region frequently becomes bending cuticle. The posterodistal region of 2Ax articulates with the PMP along the concave axillary fold line, and they are clearly divided from each other, even though the PMP is often membranous or reduced in some groups. The posterior tip of 2Ax usually forms an articulation with the anterior lobe of 3Ax, along the concave axillary fold line, but they are separated by a membrane.

The 3Ax consists of three lobes, the anterior, proximal, and distal lobes. Its central region is a plate-like sclerite. The proximal lobe articulates with the PNWP. The anterior lobe articulates with the posterior tip of 2Ax. The distal lobe articulates with the base of the anal veins. 3Ax is the active sclerite of the flexor mechanism, which directly manipulates the anal veins [20].

The PMP is a roughly triangular or trapezoid sclerite anteriorly distal to the posterior region of 2Ax and posteriorly distal to 3Ax. The sclerotization of the PMP is much weaker than that of the other axillary sclerites. The PMP is usually associated with the tip of the distal lobes of 3Ax. The DMP, whose shape is similar to but much larger than the PMP, is located distal to the PMP. The anterior margin of the DMP is always associated with the posterior margin of the radial vein along the distal axillary flexion line, and its distal margin is associated with the media and cubital veins. The two median plates form an articulation along the convex axillary fold line. The anterior margin of the DMP is delimited by the radial vein, and the median and cubital veins arise from the distal margin of the DMP. Usually, the PMP is less sclerotized than the DMP.

The humeral plate (HP) is the basal sclerite of the costal vein. The basisubcostale (BSc) represents the proximal end of the subcostal vein, and is strongly sclerotized. The basiradiale (BR) is the basal sclerite of the radial vein, and is also strongly sclerotized. It is proximally close to the anterior margin of 2Ax. The basanale (BA) is the basal sclerites of the anal veins. The tegula (Tg) is a well-defined field of sensilla trichodea and is usually elevated above the other sclerites.

### Comparative morphology of wing base structure among Neuropterida orders

#### Megaloptera (Figs. 1, 2)

Megaloptera wing base structure consists of the fundamental elements described above. Articulations and fold- and flexion-lines also preserve the plesiomorphic condition. Configurations of wing base structure show some variations between Corydalidae and Sialidae. The following three character states are apparently different from those in the outgroup taxa and the other two orders in Neuropterida.

**Figure 2:**
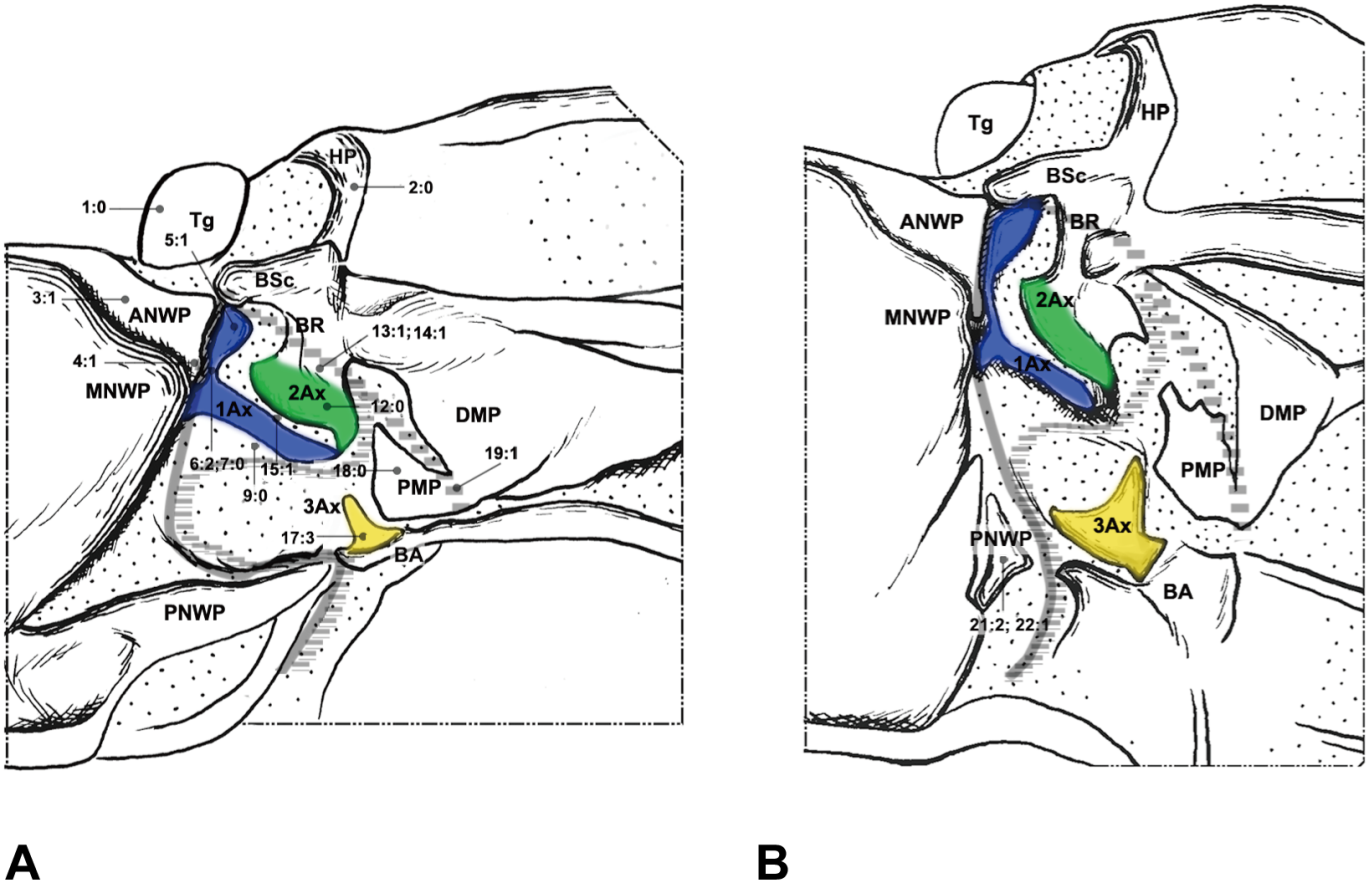
Wing base of Sialidae (Megaloptera). (A) *Sialis sibirica* McLachlan, forewing base; (B) same, hindwing base.

In Sialidae we observed a projection on the posterior corner of the ANWP (Figs. 2A, 2B), which is more obvious in the hindwing base. This projection is similar to the antemedian notal wing process (AmNWP) mentioned by Yoshizawa [29]. The character may be an autapomorphy of Sialidae in Neuropterida, but it is often present in Polyneoptera, Hemiptera, Psocoptera, and Mecoptera [29]. Therefore, the ANWP projection is probably an apomorphy of Neoptera.

The 2Ax of all investigated species has various shapes. The anterior part of 2Ax in Corydalidae bends distally (Figs. 1A, 1C), while in Sialidae, Raphidioptera and Neuroptera it bends proximally.

The PMP and DMP is fused posteriorly in Megaloptera, with the fused region becoming bending cuticle (Figs. 1A, 1C, 2A), and in the other families we examined they are separated. Fused median plates were also observed in Mantodea [29].

#### Neuroptera (Figs. 3–5)

The wing base structure of Neuroptera also retains a plesiomorphic condition. Configurations of 1Ax are quite different among the families we studied in Neuroptera. However, most of those modifications are unique for each taxon, and are regarded as derived features within Neuroptera. The detailed description is shown in result: Character description of wing base structures used for phylogenetic analysis. The following apomorphic features of Neuroptera were not observed in the outgroups, Megaloptera and Raphidioptera.

**Figure 3:**
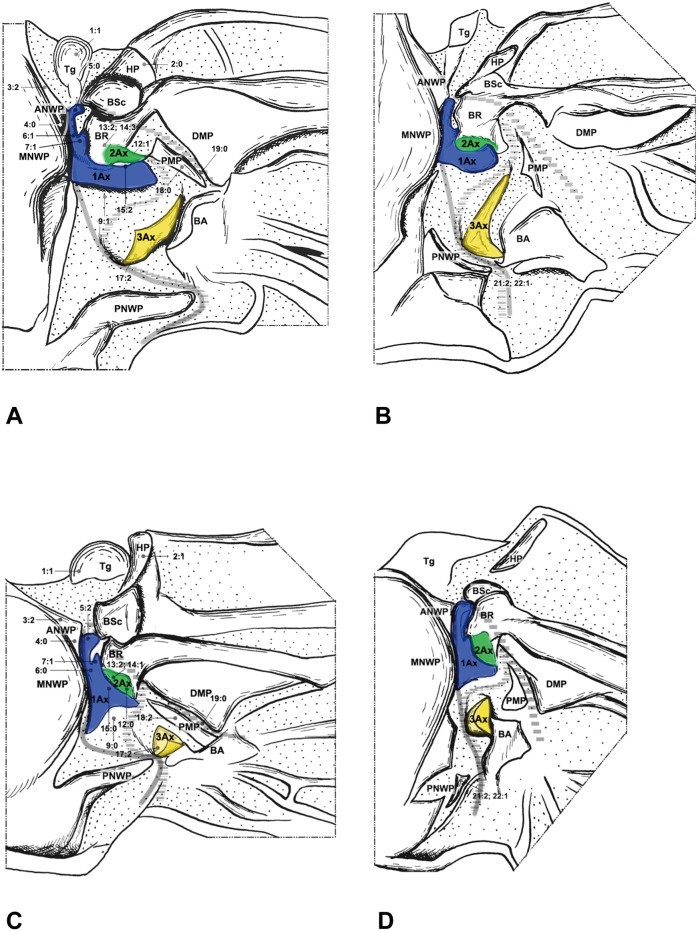
Wing base of Neuroptera. (A) *Paraglenurus japonicas* (Mclachlan) (Myrmeleontidae), forewing base; (B) same, hindwing base; (C) *Heterosmylus wolonganus* Yang (Osmylidae), forewing base; (D) same, hindwing base.

**Figure 4:**
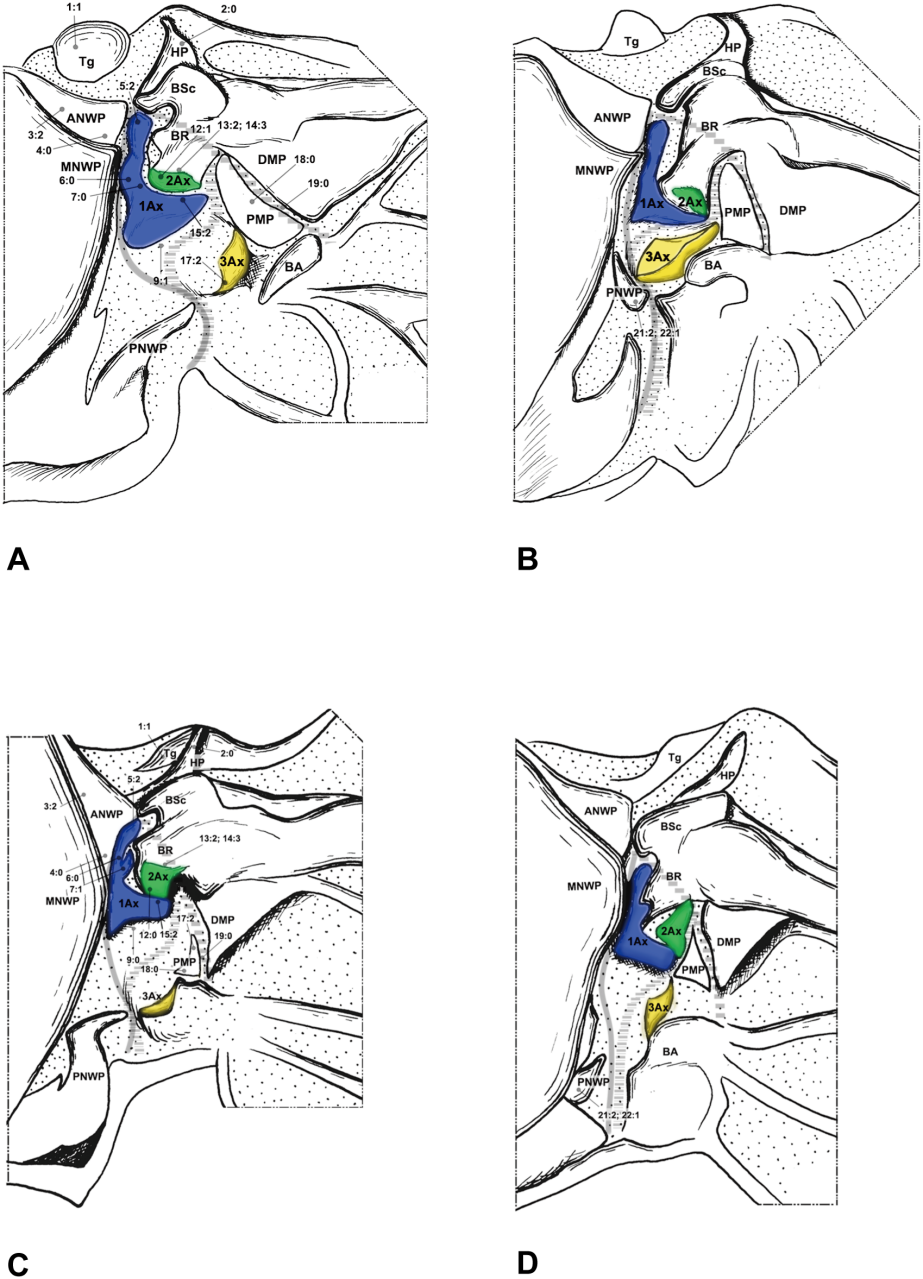
Wing base of Neuroptera. (A) *Eumantispa harmandi* (Navás) (Mantispidae), forewing base; (B) same, hindwing base; (C) *Chrysoperla* sp. (Chrysopidae), forewing base; (D) same, hindwing base.

**Figure 5:**
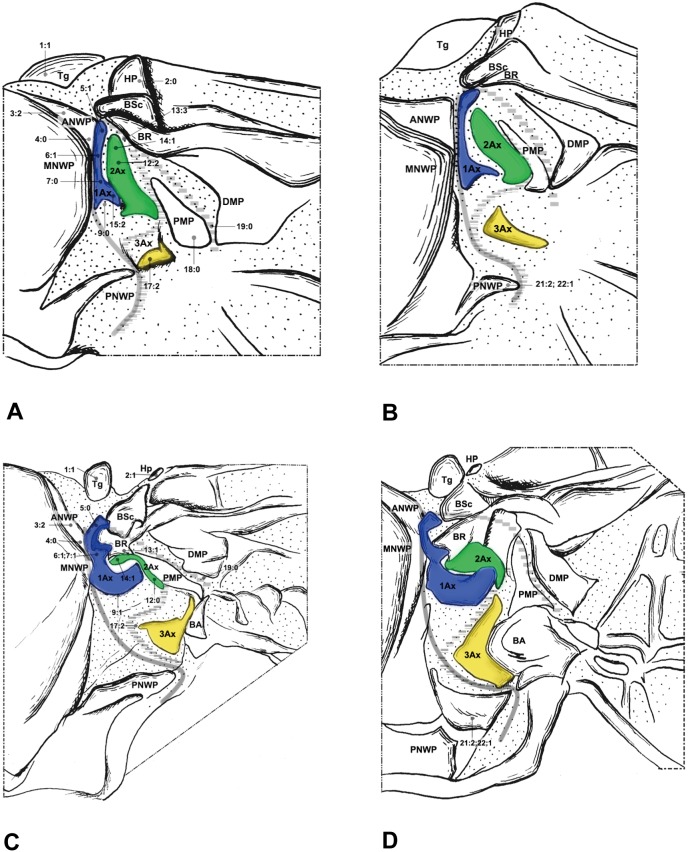
Wing base of Neuroptera. (A) *Semidalis aleyrodiformis* (Stephens) (Coniopterygidae), forewing base; (B) same, hindwing base; (C) *Sulphalasca* sp. (Ascalaphidae), forewing base; (D) same, hindwing base.

The HP of Neuroptera is a detached sclerite (Figs. 3A, 3C, 4A, 4C, 5A, 5C). In contrast, it is fused to Costa and BSc in the remaining groups we examined. The detached HP may be a potentially derived character in Neoptera.

**Figure 6:**
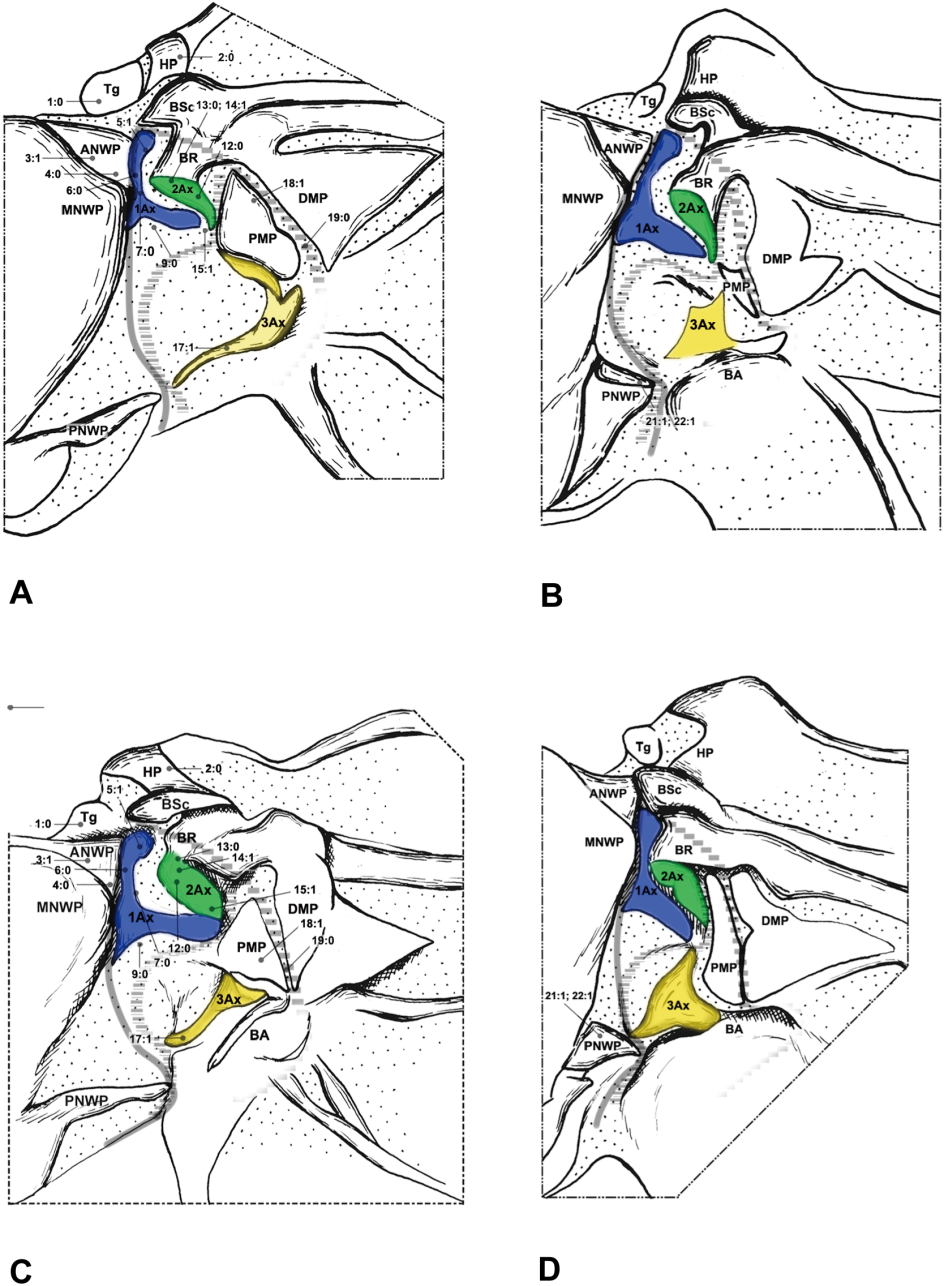
Wing base of Raphidioptera. (A) *Inocellia fujiana* Yang (Inocelliidae), forewing base; (B) same, hindwing base; (C) *Xanthostigma gobicola* Aspöck & Aspöck (Raphidiidae), forewing base; (D) same, hindwing base.

The stripe-like ANWP (Figs. 3A, 3C, 4A, 4C, 5A, 5C) is always observed in Neuroptera. However, in Megaloptera and Raphidioptera, it is normally triangular (Figs. 1A, 1C, 2A, 6A, 6C). This phenomenon is concordant with observations by Beutel et al. [5].

In Neuroptera, 2Ax is always fused to the BR (Figs. 3A, 3C, 4A, 4C, 5A, 5C). Apical fusion of 2Ax with the BR is observed in Corydalidae and Raphidioptera. In Sialidae, the anterodistal part of 2Ax is fused to the BR. The BR and 2Ax are separated only in Coniopterygidae, which may be an autapomorphy of Coniopterygidae.

#### Raphidioptera (Fig. 6)

The wing base structure in Raphidioptera bears a rather primitive condition in the shapes of the sclerites, articulations, and fold and flexion lines. Therefore, the homology of each structure is rather easily determined. The following features, which are different from Megaloptera, Neuroptera, and the outgroups, are observed throughout Raphidioptera.

**Figure 7:**
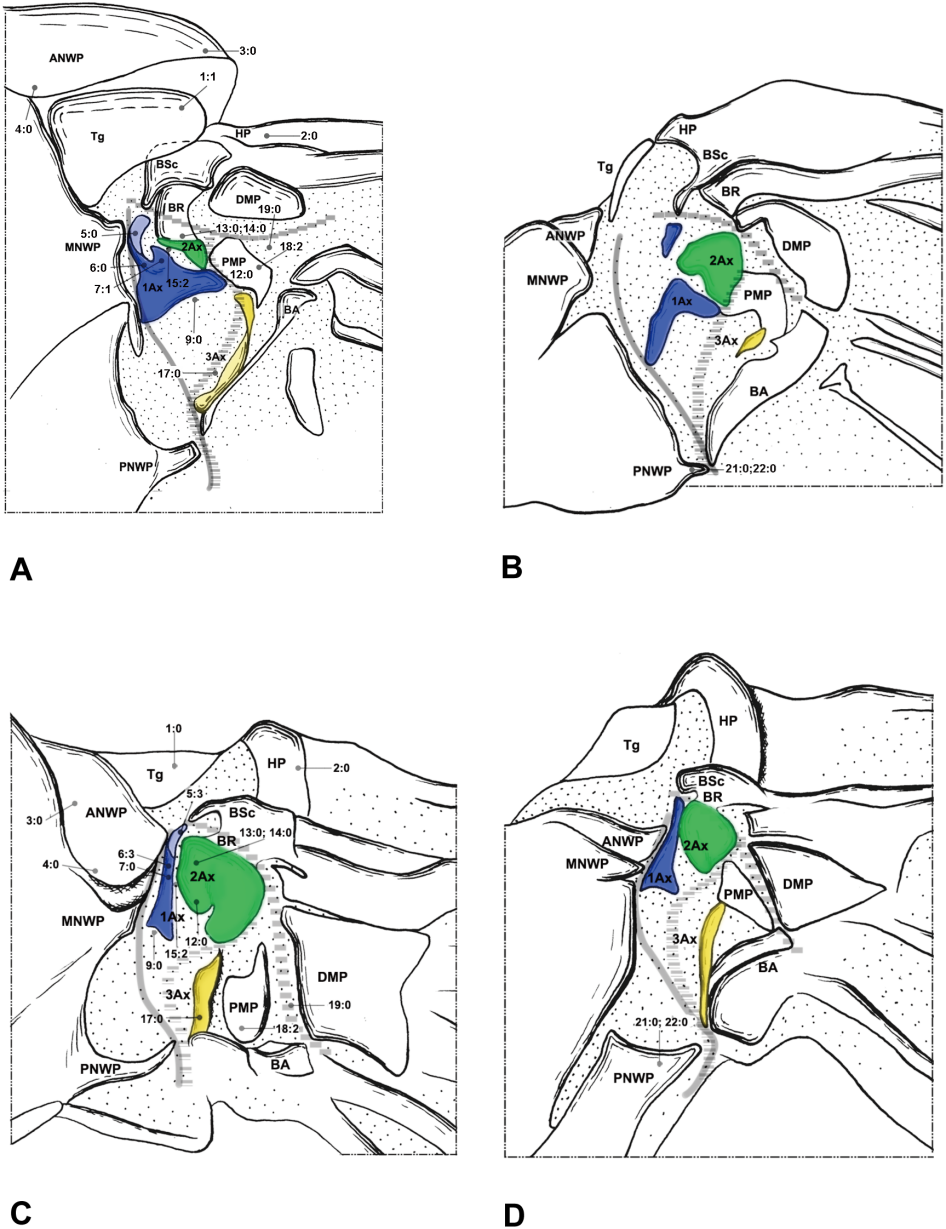
Wing base of Hymenoptera. (A) *Tenthredo* sp. (Tenthredinidae), forewing base; (B) same, hindwing base. Wing base of Psocoptera. (C) *Taeniostigminae* sp. (Amphipsocidae), forewing base; (D) same, hindwing base.

In Raphidioptera, the angle between the distal margins of the body and neck of 1Ax is between 80° and 100°. In Megaloptera and Neuroptera, the angle is between 110° and 130°. In the outgroups (Fig. 7), the distal margins of the neck and body are nearly straight (∼180°).

The neck of 1Ax lacks any projection in Raphidioptera. In some families of Megaloptera (Figs. 1A, 1C) and Neuroptera (Figs. 3A, 3C, 4C), a projection set off from the bottom of the 1Ax neck was observed, and this projection can also help distinguish the head, neck, and body of 1Ax. This projection was not observed in Polyneoptera [29] and Hemiptera [39], but can be observed in Mecoptera [5].

## Character Description of Wing Base Structures Used for Phylogenetic Analysis

### Forewing

**Figure 8:**
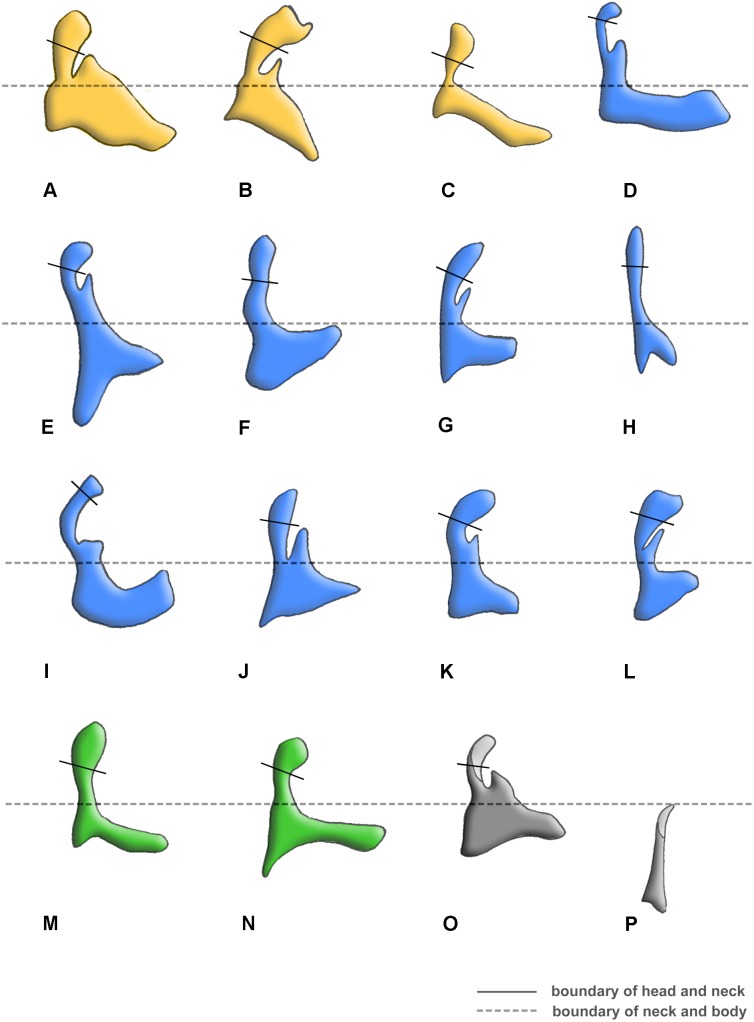
Morphological variations of 1Ax among Neuropterida families. (A) *Protohermes costalis* (Walker) (Corydalinae); (B) *Neochauliodes punctatolosus* Liu & Yang (Chauliodinae); (C) *Sialis sibirica* McLachlan (Sialidae); (D) *Paraglenurus japonicas* (McLachlan) (Myrmeleontidae); (E) *Heterosmylus wolonganus* Yang (Osmylidae); (F) *Eumantispa harmandi* (Navás) (Mantispidae); (G) *Chrysoperla* sp. (Chrysopidae); (H) *Semidalis aleyrodiformis* (Stephens) (Coniopterygidae); (I) *Sulphalasca* sp. (Ascalaphidae); (J) *Dilar hastatus* Zhang, Liu, Aspöck & Aspöck (Dilaridae); (K) *Nipponeurorthus fuscinervis* Nakahara (Nevrorthidae); (L) *Hemerobius* sp. (Hemerobiidae); (M) *Inocellia fujiana* Yang (Inocelliidae); (N) *Xanthostigma gobicola* Aspöck & Aspöck (Raphidiidae); (O) *Tenthredo* sp. (Hymenoptera: Tenthredinidae); (P) *Taeniostigminae* sp. (Psocoptera: Amphipsocidae).

1. Tegula: (0) membranous; (1) sclerotized. We separated the states by applying force to the Tegula. The sclerotized tegula was observed in Neuroptera (Figs. 3A, 3C, 4A, 4C, 5A, 5C) (CI = 0.50, RI = 0.80).
2. HP: (0) fused to Costa and BSc; (1) separated from Costa and BSc. The detached HP was observed in Neuroptera (Figs. 3A, 3C, 4A, 4C, 5A, 5C). (CI = 1.00, RI = 1.00).
3. Shape of ANWP: (0) Not triangular or stripe-like; (1) triangular; (2) stripe-like. State 1 which is also mentioned in Friedrich & Beutel [42] was observed in Megaloptera (Figs. 1A, 1C, 2A) and Raphidioptera (Figs. 6A, 6C), and state 2 was observed in Neuroptera (Figs. 3A, 3C, 4A, 4C, 5A, 5C). (CI = 1.00, RI = 1.00).
4. Posterodistal projection of ANWP: (0) absent; (1) present. The posterodistal projection of ANWP is present in Sialidae (Fig. 2A), which may be an autapomorphy of this family. (CI = 1.00, RI = 1.00).
5. Width of head of 1Ax: (0) narrower than neck; (1) wider than neck; (2) almost as wide as neck; (3) absent. This is a quantitative character, but there was no ambiguity separating two states of this character. State 1 was observed in Megaloptera (Figs. 8A–C) and Raphidioptera (Figs. 8M–N). State 2 was observed in Neuroptera (Figs. 8D–L). In Psocoptera, head-neck region of 1Ax is almost absent [47]. (CI = 0.33, RI = 0.14).
6. Length of neck of 1Ax: (0) as long as the head of 1Ax; (1) two times as long as the head of 1Ax; (2) half the length of the head of 1Ax; (3) absent. State 1 was observed in Coniopterygidae (Fig. 5A), Ascalaphidae (Fig. 5C) and Myrmeleontidae (Fig. 3A). State 2 was observed in Megaloptera (Figs. 1A, 1C, 2A). (CI = 0.33, RI = 0.45).
7. Neck of 1Ax: (0) without projections; (1) with a projection set off from the bottom of neck; (2) absent. State 1 was observed in Corydalidae (Figs. 1A, 1C), Myrmeleontidae (Fig. 3A), Osmylidae (Fig. 3C), Chrysopidae (Fig. 4C), Ascalaphidae (Fig. 5C), Dilaridae, Nevrorthidae and Hemerobiidae. (CI = 0.33, RI = 0.20).
8. Body of 1Ax: (0) triangular; (1) rectangular. State 1 was observed in Myrmeleontidae and Ascalaphidae (Figs. 3A, 5C). (CI = 1.00, RI = 1.00).
9. Posterior margin of body of 1Ax: (0) concave; (1) convex. State 1 was observed in Myrmeleontidae (Fig. 3A), Ascalaphidae (Fig. 5C), Hemerobiidae, Nevrorthidae and Mantispidae (Fig. 4A). (CI = 0.25, RI = 0.25).
10. Transition from body to neck in 1Ax: (0) recognized by abrupt change of width or projection; (1) hardly recognized owing to absence of neck. State 1 was observed in Amphipsocidae (Fig. 7C). (CI = 1.00, RI = 1.00).
11. Angle between distal margin of body and neck of 1Ax: (0) not as for state (1) or (2); (1) between 110° and 130°; (2) between 80° and 100°. State 1 was observed in Neuroptera and Megaloptera (Figs. 1A, 1C, 2A, 3A, 3C, 4A, 4C, 5A, 5C). State 2 was observed in Raphidioptera (Figs. 6A, 6C). (CI = 0.66, RI = 0.66).
12. Size of 2Ax: (0) almost as long as distal lobe of body of 1Ax; (1) at most half the length of the distal lobe of body of 1Ax; (2) larger than 1Ax. State 1 was observed as a synapomorphy of Myrmeleontidae (Fig. 3A) and Mantispidae (Fig. 4A). Although, in Neuroptera, except for Coniopterygidae, 2Ax is fused to BR completely, we can recognize its general size by combining the darker region and sclerotization. State 2 was observed as an autapomorphy of Coniopterygidae (Fig. 5A). (CI = 0.50, RI = 1.00).
13. Contact between BR and 2Ax: (0) 2Ax partly fused to BR; (1) 2Ax fused to BR completely; (2) separated. State 0 was observed in Megaloptera (Figs. 1A, 1C, 2A) and state 1 was observed in Neuroptera (Figs. 3A, 3C, 4A, 4C, 5A, 5C). State 2 was observed probably as an autapomorphy in Coniopterygidae (Fig. 5A). (CI = 1.00, RI = 1.00).
14. Anterior part of 2Ax: (0) bends cranially; (1) bends proximally; (2) bends distally. State 1 was observed in Raphidioptera, Neuroptera (Figs. 3A, 3C, 4A, 4C, 5A, 5C, 6A, 6C) and Coniopterygidae (Fig. 5A), while state 2 was observed in some Megaloptera (Figs. 1A, 1C). (CI = 1.00, RI = 1.00).
15. Articulation between 1Ax and 2Ax: (0) along proximal margin of 2Ax; (1) at proximo-caudal point of 2Ax; (2) at proximo-cranial point of 2Ax. State 1 was observed in Raphidioptera and Sialidae (Figs. 2A, 6A, 6C) and state 2 was observed in Amphipsocidae (Fig. 7C). (CI = 0.66, RI = 0.80).
16. Number of lobes of 3Ax: (0) three; (1) two. State 1 was observed in outgroups (Figs. 7A, 7C). (CI = 1.00, RI = 1.00).
17. Size of lobes of 3Ax: (0) two times as long as wide; (1) more than three times as long as wide; (2) as long as wide; (3) two times as wide as long. Because 3Ax is rotated, it may look different from certain angles in different specimens of the same species. State 1 was observed in Raphidioptera (Figs. 6A, 6C). State 2 was observed in Neuroptera (Figs. 3A, 3C, 4A, 4C, 5A, 5C). State 3 was observed in Megaloptera (Figs. 1A, 1C, 2A). (CI = 1.00, RI = 1.00).
18. PMP: (0) not reduced and with same degree of sclerotization with DMP; (1) not reduced but less sclerotized than DMP; (2) reduced or membranous compared to DMP. State 1 was observed in Raphidioptera (Figs. 6A, 6C), and state 2 was observed in Neuroptera (Figs. 3A, 3C, 4A, 4C, 5A, 5C). (CI = 0.66, RI = 0.83).
19. DMP and PMP: (0) separated; (1) fused posteriorly. State 1 was observed in Megaloptera (Figs. 1A, 1C, 2A). (CI = 1.00, RI = 1.00).

### Hindwing

20. Neck of 1Ax: (0) present; (1) absent. State 1 was observed in Tenthredinidae (Fig. 7B). (CI = 1.00, RI = 1.00).
21. Shape of detached part of PNWP: (0) absent; (1) triangular; (2) not triangular. State 1 was observed in Raphidioptera (Figs. 6B, 6D). (CI = 1.00, RI = 1.00).
22. PNWP: (0) without detached sclerite; (1) with a detached sclerite. State 1 is frequently present in hindwing and was observed in Neuropterida (Figs. 3B, 3D, 4B, 4D, 5B, 5D). (CI = 0.25, RI = 0.25).
23. PNWP: (0) sclerotized; (1) less sclerotized. We separated these two states by applying force to the surface. If its sclerotization is the same as 1Ax, we described it as sclerotized. If its sclerotization is the same as DMP, we described it as less sclerotized. State 1 was observed in Megaloptera (Figs. 1B, 1D, 2B) and Neuroptera (Figs. 3B, 3D, 4B, 4D, 5B, 5D). (CI = 1.00, RI = 1.00).

## Phylogenetic Analyses

**Figure 9:**
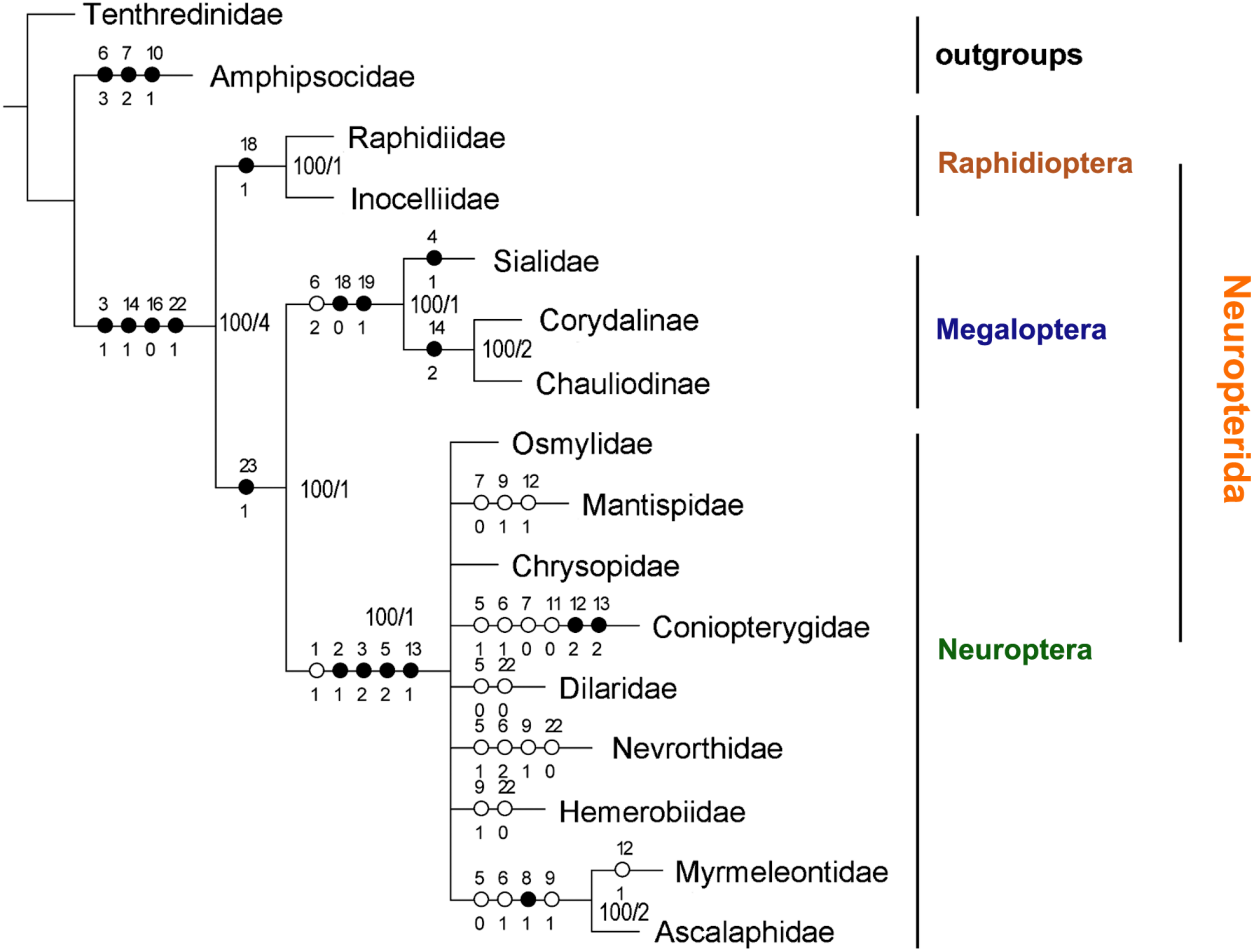
Higher taxa Neuropterida phylogeny inferred from wing base data. A strict consensus of the most parsimonious trees inferred from the fore- and hindwing base data are presented. Only unambiguous characters are mapped. Filled circles represent homologous characters, open circles represent reversal or parallel characters. Character states are placed below the circles. Numbers on nodes indicate the bootstrap values and Bremer’s decay indices.

Our matrix analysis yielded 41 equally most parsimonious trees (MPT) (tree length = 57; CI = 0.66; RI = 0.73). Topologies of the 41 MPTs are different only in the relationships within the Neuroptera; their strict consensus tree is shown in Fig. 9. In addition, PAUP*4.0b10 yielded the same results. The monophyly of Neuropterida was well supported by four homologous apomorphies: (1) the triangular ANWP (char. 3∶1), (2) the anterior part of 2Ax bends proximally (char. 14∶1), (3) 3Ax has three lobes (char. 16∶0), and (4) the PNWP has a detached sclerite (char. 22∶1). Neuroptera was assigned as the sister of Megaloptera, on the basis of the homologous synapomorphy that both have a less sclerotized detached part of the hind wing PNWP (char. 23∶1). The monophyly of Megaloptera was supported by three synapomorphies: (1) a 1Ax short neck (half the length of the head) (char. 6∶2), (2) the same strong sclerotization of the PMP and DMP (char. 18∶0), and (3) the fusion of the posterior part of the DMP and PMP (char. 19∶1). The monophyly of Neuroptera was supported by four homologous synapomorphies: (1) the detached HP (char. 2∶1), (2) the strip ANWP (char. 3∶2), (3) the width of the head of 1Ax (almost as wide as the neck) (char. 5∶2), and (4) 2Ax completely fused to the BR (char. 13∶1). The monophyly of Raphidioptera was supported by one homologous synapomorphy: the PMP is not reduced, but is less sclerotized than the DMP (char. 18∶1).

Additionally, in thirty-nine MPTs Conioterygidae and the remaining neuropteran families were assigned as sister groups, which is concordant with the result in Winterton et al. [18] based on combined multi-gene analysis with morphological data. However, in the strict consensus tree the sister relationship mentioned above collapsed because of a low nodal support value. The sister group relationship between Ascalaphidae and Myrmeleontidae was corroborated in all MPTs as well as in the strict consensus tree, and it is consistent with the general viewpoint that these two families form a monophylum [10], [18]. In our study, the single homologous synapomorphy supporting Ascalaphidae + Myrmeleontidae was the rectangular body shape of 1Ax (char. 8∶1) in both.

## Discussion

Our phylogenetic analysis of fore- and hindwing base structural data support the monophyly of Megaloptera and a sister group relationship between Megaloptera and Neuroptera, which is consistent with many results based on morphology and mitochondrial genomic data [8], [10], [12], [13], as well as the very recent phylogenetic analysis based on transcriptome and comprehensive morphological data [40]. Furthermore, the traditional higher classification within Megaloptera, (i.e. Sialidae + (Corydalinae + Chauliodinae)) was corroborated based on our wing base data. Beutel et al. [5] suggested a monophyletic Neuropterida based on thoracic characters, mainly thoracic sclerites and muscle structure. They assigned Megaloptera and Raphidioptera to a monophylum, in which Megaloptera is paraphyletic to Corydalidae, being the sister of Raphidioptera. Beutel et al. [5] also argued that the monophyly of the Raphidioptera–Megaloptera lineage appears very likely, considering consistent support from several independent analyses based on extensive morphological and molecular data sets. They further suggested that the proposed Neuroptera–Megaloptera sister-group relationship was not convincing, although only one representative of each of the three Neuropterida orders was included in the study [12]. Nevertheless, recent molecular phylogenomic studies based on a more comprehensive sampling of Neuropterida [13], [41] support the sister relationship between Megaloptera and Neuroptera. Furthermore, the well accepted view that Neuropterida and Coleoptera form a monophylum was not supported in Friedrich and Beutel [42] and Beutel et al. [5] using a large number of thoracic characters, but without any wing base data. Friedrich and Beutel [42] also considered the Corydalidae–Raphidioptera clade to be an artifact, caused by the advanced predatory larval habits of the two groups resulting in head structure modification [43]. Therefore, the suitability of using thoracic- and muscle-based characters to address the interordinal phylogeny of Neuropterida needs to be reconsidered.

The wing base contains a complicated arrangement of several sclerites. Among the representative taxa of Megaloptera and Raphidioptera that we examined, those species from Corydalidae were large-sized, with a forewing length of ∼50 mm, and had the strongest and the most complex wing base structure; while in other relatively smaller species, that were not Corydalidae, the wing base appears much weaker and simpler. Although Megaloptera and Raphidioptera have poor long-distance dispersal capacity [44], [45], Corydalidae generally have a better flight capability than Sialidae and Raphidioptera [3], [46], seemingly suggesting that the complexity of the wing base is somewhat correlated to the capacity of flight, as those insects with the larger body size and better flight ability have a stronger and more complex wing base structure. However, the wing base complexity between large-sized and small-sized species, as well as between strong and poor neuropteran fliers could not be studied here because of limited sample availability. Nonetheless, the morphological derivation of the wing base correlated to flight ability appears to have no obvious bias in the present phylogenetic analysis.

The wing base is a useful, but largely ignored, structure for reconstructing the phylogeny of the Neuropterida. Recent published works on the phylogenetic reconstruction of some insect groups, e.g. Paraneopteran orders [34], Zoraptera [28], and Polyneoptera [29], all based on wing base data, mainly recovered higher phylogenetic relationships among the orders. In our study, although the relationships among the three orders of Neuropterida were clearly resolved, the interfamilial relationships within Neuroptera had poor resolution, with only Ascalaphidae and Myrmeleontidae being assigned as a sister group. It is obvious that the configuration of the wing base (e.g. the forewing 1Ax, Fig. 8) among families of Neuroptera is variable, but there were an insufficient number of informative characters available to solve the interfamilial phylogeny in our present study. The geometric morphometrics of these wing base sclerites may allow further resolution of the interfamilial phylogeny in a future comprehensive study.

## Supporting Information

S1 Table Taxa examined.
(DOC)

S2 Table Data matrix for the present phylogenetic analysis.
(DOCX)
