# Supplementary material for: Wing Base Structural Data Support the Sister Relationship of Megaloptera and Neuroptera (Insecta: Neuropterida)
Source: PLoS One. 2014 Dec 11;9(12):e114695. doi: 10.1371/journal.pone.0114695 (PMC4263614; doi:10.1371/journal.pone.0114695)
Supplement: S1 Table — Taxa examined. (DOC) [file pone.0114695.s001.doc]

**Table 1. Taxa examined.**

| **Taxon** | **Specimen** | **Collection information** |
| --- | --- | --- |
| Megaloptera |  |  |
| **Corydalidae** |  |  |
| Corydalinae |  |  |
| *Protohermes costalis* (Walker) | 2♂ | CHINA, Zhejiang, Lin-an, Tianmushan, (CAU) |
| Chauliodinae |  |  |
| *Neochauliodes punctatolosus* Liu & Yang | 2♂ | CHINA, Yunnan, Xishuangbanna, Menglun,2009.V.30, Xiushuai Yang (CAU). |
| **Sialidae** |  |  |
| *Sialis sibirica* McLachlan | 2♀ | CHINA, Heilongjiang, Maoershan, 2011.VII.2, Junchao Wang (CAU). |
| Neuroptera |  |  |
| **Myrmeleontidae** |  |  |
| *Paraglenurus japonicas* (McLachlan) | 2♀ | CHINA, Jiangxi, Wuyishan, 2012.VIII.11, Guoquan Wang (CAU). |
| **Osmylidae** |  |  |
| *Heterosmylus wolonganus* Yang | 1♂ | CHINA, Henan, Songxian, Baiyunshan,  2008.III.15, Weihai Li (CAU). |
| *Thyridosmylus* sp. | 1♀ | CHINA, Tibet, Hanmi, 2011.VIII.6, Lihua Wang (CAU). |
| **Mantispidae** |  |  |
| *Eumantispa harmandi* (Navás) | 1♀ | CHINA, Hebei, Wulingshan 2008.IX.6, Qifei Liu (CAU). |
| *Mantispa* sp. | 1♀ | CHINA, Tibet, Tongmai, 2011.VIII.21, Lihua Wang (CAU). |
| **Chrysopidae** |  |  |
| *Chrysoperla* sp. | 1♀ | CHINA, Sichuan, Emeishan, 2012.VIII.20, Liang Wang (CAU). |
| *Italochrysa* sp. | 1♀ | CHINA, Sichuan, Emeishan, 2012.VIII.20, Liang Wang (CAU). |
| **Coniopterygidae** |  |  |
| *Semidalis aleyrodiformis* (Stephens) | 2♂ | CHINA, Beijing, Campus of China Agricultural University, 2011.VIII.26, Junchao Wang, (CAU). |
| **Ascalaphidae** |  |  |
| *Sulphalasca* sp. | 1♀ | CHINA, Taiwan, Dahanshan, 2011.VI.7, Xiaoyan Liu (CAU). |
| *Sulphalomitus formosanus* Esben-Petersen | 1♀ | CHINA, Taiwan, Dahanshan, 2011.VI.7, Xiaoyan Liu (CAU). |
| **Dilaridae** |  |  |
| *Dilar hastatus* Zhang, Liu, Aspöck & Aspöck | 2♂ | CHINA, Beijing, Mentougou, Xiaolongmen,  2012.VI.23, Xuankun Li (CAU). |
| **Nevrorthidae** |  |  |
| *Nipponeurorthus fuscinervis* (Nakahara) | 1♀ | JAPAN, Hokkaido， Honbetsu, Yusen-kyo，  2013.VII.1, Xingyue Liu (CAU). |
| **Hemerobiidae** |  |  |
| *Hemerobius* sp. | 2♂ | JAPAN, Hokkaido, Ashoro, Onneto,  2013.VI.30, Xingyue Liu (CAU). |
| Raphidioptera |  |  |
| **Raphidiidae** |  |  |
| *Xanthostigma gobicola* Aspöck & Aspöck | 2♂ | CHINA, Beijing, Yanqing, Songshan,  2010.VI.3, Lihua Wang (CAU). |
| **Inocelliidae** |  |  |
| *Inocellia fujiana* Yang | 2♂ | CHINA, Shanxi, Taigu, (CAU). |
| Hymenoptera |  |  |
| **Tenthredinidae** |  |  |
| *Tenthredo* sp. | 2♀ | CHINA, Neimenggu, Saihanwula, Dadonggou, 2013.VII.24, Xiumei Lu (CAU). |
| Psocoptera |  |  |
| **Amphipsocidae** |  |  |
| *Taeniostigminae* sp. | 2♀ | CHINA, Chongqing, Liangping, Dongshanlinchang,  2012.VIII.14, Zhifei Li (CAU). |
