# Supplementary material for: Wing Base Structural Data Support the Sister Relationship of Megaloptera and Neuroptera (Insecta: Neuropterida)
Source: PLoS One. 2014 Dec 11;9(12):e114695. doi: 10.1371/journal.pone.0114695 (PMC4263614; doi:10.1371/journal.pone.0114695)
Supplement: S2 Table — Data matrix for the present phylogenetic analysis. (DOCX) [file pone.0114695.s002.docx]

**Table 2. Data matrix for present phylogenetic analysis**

|  |  |  |  |  |  |  |  |  |  | 1 | 1 | 1 | 1 | 1 | 1 | 1 | 1 | 1 | 1 | 2 | 2 | 2 | 2 |
| --- | --- | --- | --- | --- | --- | --- | --- | --- | --- | --- | --- | --- | --- | --- | --- | --- | --- | --- | --- | --- | --- | --- | --- |
|  | 1 | 2 | 3 | 4 | 5 | 6 | 7 | 8 | 9 | 0 | 1 | 2 | 3 | 4 | 5 | 6 | 7 | 8 | 9 | 0 | 1 | 2 | 3 |
| Tenthredinidae | 1 | 0 | 0 | 0 | 0 | 0 | 0 | 0 | 0 | 0 | 0 | 0 | 0 | 0 | 2 | 1 | 0 | 2 | 0 | 1 | 0 | 0 | 0 |
| Amphipsocidae | 0 | 0 | 0 | 0 | 3 | 3 | 2 | 0 | 0 | 1 | 0 | 0 | 0 | 0 | 2 | 1 | 0 | 2 | 0 | 0 | 0 | 0 | 0 |
| Corydalinae | 0 | 0 | 1 | 0 | 1 | 2 | 1 | 0 | 0 | 0 | 1 | 0 | 0 | 2 | 0 | 0 | 3 | 0 | 1 | 0 | 2 | 1 | 1 |
| Chauliodinae | 0 | 0 | 1 | 0 | 1 | 2 | 1 | 0 | 0 | 0 | 1 | 0 | 0 | 2 | 0 | 0 | 3 | 0 | 1 | 0 | 2 | 1 | 1 |
| Sialidae | 0 | 0 | 1 | 1 | 1 | 2 | 0 | 0 | 0 | 0 | 1 | 0 | 0 | 1 | 1 | 0 | 3 | 0 | 1 | 0 | 2 | 1 | 1 |
| Myrmeleontidae | 1 | 1 | 2 | 0 | 0 | 1 | 1 | 1 | 1 | 0 | 1 | 1 | 1 | 1 | 0 | 0 | 2 | 2 | 0 | 0 | 2 | 1 | 1 |
| Osmylidae | 1 | 1 | 2 | 0 | 2 | 0 | 1 | 0 | 0 | 0 | 1 | 0 | 1 | 1 | 0 | 0 | 2 | 2 | 0 | 0 | 2 | 1 | 1 |
| Mantispidae | 1 | 1 | 2 | 0 | 2 | 0 | 0 | 0 | 1 | 0 | 1 | 1 | 1 | 1 | 0 | 0 | 2 | 2 | 0 | 0 | 2 | 1 | 1 |
| Chrysopidae | 1 | 1 | 2 | 0 | 2 | 0 | 1 | 0 | 0 | 0 | 1 | 0 | 1 | 1 | 0 | 0 | 2 | 2 | 0 | 0 | 2 | 1 | 1 |
| Coniopterygidae | 1 | 1 | 2 | 0 | 1 | 1 | 0 | 0 | 0 | 0 | 1 | 2 | 2 | 1 | 0 | 0 | 2 | 2 | 0 | 0 | 2 | 1 | 1 |
| Ascalaphidae | 1 | 1 | 2 | 0 | 0 | 1 | 1 | 1 | 1 | 0 | 1 | 0 | 1 | 1 | 0 | 0 | 2 | 2 | 0 | 0 | 2 | 1 | 1 |
| Dilaridae | 1 | 1 | 2 | 0 | 0 | 0 | 1 | 0 | 0 | 0 | 1 | 0 | 1 | 1 | 0 | 0 | 2 | 2 | 0 | 0 | 2 | 0 | 1 |
| Nevrorthidae | 1 | 1 | 2 | 0 | 1 | 2 | 1 | 0 | 1 | 0 | 1 | 0 | 1 | 1 | 0 | 0 | 2 | 2 | 0 | 0 | 2 | 0 | 1 |
| Hemerobiidae | 1 | 1 | 2 | 0 | 2 | 0 | 1 | 0 | 1 | 0 | 1 | 0 | 1 | 1 | 0 | 0 | 2 | 2 | 0 | 0 | 2 | 0 | 1 |
| Raphidiidae | 0 | 0 | 1 | 0 | 1 | 0 | 0 | 0 | 0 | 0 | 2 | 0 | 0 | 1 | 1 | 0 | 1 | 1 | 0 | 0 | 1 | 1 | 0 |
| Inocelliidae | 0 | 0 | 1 | 0 | 1 | 0 | 0 | 0 | 0 | 0 | 2 | 0 | 0 | 1 | 1 | 0 | 1 | 1 | 0 | 0 | 1 | 1 | 0 |
